# Synergistic Activity of APOL3 and GBP1 in Antigen Cross-Presentation: A Model

**DOI:** 10.3390/biom16101387

**Published:** 2026-09-23

**Authors:** Etienne Pays

**Affiliations:** Institute of Molecular Biology and Medicine, University of Brussels, B6041 Gosselies, Belgium; etienne.pays@gmail.com

**Keywords:** antigen cross-presentation, apolipoproteins L, apoptosis, guanylate-binding protein, infection, inflammation, innate immunity, membrane megapores, parasitophorous vacuole, phagolysosomal membrane

## Abstract

Antigen cross-presentation by dendritic cells allows the induction of an immune response (activation of cytotoxic CD8^+^ T cells) against internalized components from pathogens or dying tumour cells, through export of these components from the lumen of phagosomes to the cell surface. Such export requires megapore formation in the phagolysosomal membrane, as also occurs in the outer membrane of mitochondria for cytochrome c export during apoptosis. Two interferon-induced proteins synergistically participate in this process: apolipoprotein L3 (APOL3) (or its murine homologue mAPOL7C) and the dynamin-like GTPase guanylate-binding protein-1 (GBP1). In this Perspective, it is proposed that phagolysosomal membrane disorganization by GBP1 allows pore formation by APOL3, through transmembrane insertion and cardiolipin solubilization. Moreover, both proteins could induce activation of non-muscle myosin 2A (NM2A) and pro-apoptotic BCL2-associated protein X (BAX), which allow phagolysosomal and mitochondrial pore expansion and stabilization into open megapores. Given the potential of its transmembrane α-helices for oligomerization, APOL3 may participate in the inner coating of phagolysosomal membrane megapores.

## 1. Introduction: Phospholipid-Dependent Membrane Remodelling Linked to Infection

Cell infection by various pathogens (bacteria, viruses or parasites) can induce intensive endomembrane remodelling programmed by the invaders to favour their growth, but the immune response also triggers membrane rearrangements. In both cases, membrane changes heavily rely on phosphoinositide (PI) dynamics [1]. Among these changes, induction of inflammation involves the traffic of Golgi-derived vesicles carrying the autophagy 9A (ATG9A) lipid scramblase and the phosphatidylinositol-4-phosphate (PI4P)-synthesizing phosphatidylinositol 4-kinase III β (PI4KB) to reach mitochondrion–endoplasmic reticulum (ER) contact sites (MERCs) for auto/mitophagy initiation [2,3], together with activation, then degradation, of the inflammation factor STING (stimulator of interferon genes) [4,5]. In both processes, PI4P synthesis by PI4KB is required for membrane remodelling. Through its ability to recruit various membrane shaping and trafficking agents, PI4P is a key player for vesicle formation, traffic, and fusion [2,3,6,7,8,9,10,11,12].

Inflammation is initiated in antigen-presenting cells such as dendritic cells (DCs) and macrophages, which are alerted by danger signals for engulfment and processing of pathogen components in a vacuole termed the phagolysosome [13]. Similarly, dying tumour cells can be internalized in phagolysosomal membranes [14]. As for vesicular dynamics, the formation and maturation of the phagolysosome crucially depend on PI4P synthesis [15]. Pathogens that can survive and grow in phagolysosomes—such as the intracellular bacteria *Legionella*, *Mycobacterium*, *Chlamydia*, or *Salmonella*, or the intracellular parasites *Leishmania*, *Plasmodium*, *Toxoplasma*, *Eimeria*, or *Cryptosporidium*—develop in a vacuole termed the pathogen-containing vacuole or parasitophorous vacuole (PV) [16,17]. However, internalized pathogen factors can be recycled to the DC surface to activate CD8^+^ cytotoxic T lymphocytes, in a process known as antigen cross-presentation [18].

Antigen crossing from the PV lumen involves the formation of membrane pores likely to be larger than ionic pores. Indeed, the transmembrane pores required for antigen cross-presentation must be bigger than standard ionic channels to allow macromolecules such as proteins or DNA to escape the phagolysosome. Such observation is reminiscent of mitochondrial membrane megapores linked to apoptosis, which depend on the diphospholipid cardiolipin to allow cytochrome c to cross through the outer organelle membrane [19]. Similarly, apoptotic-like membrane megapores may be needed in the phagolysosomal membrane for antigen cross-presentation at the surface of DCs.

## 2. Synergistic Activity of Apolipoprotein L3 (APOL3) and Guanylate-Binding Protein 1 (GBP1) in Antigen Cross-Presentation

Human APOL3, or its putative murine homologue mAPOL7C, is an endomembrane-associated protein strongly induced by Toll-like receptor 3 (TLR3) signalling in DCs and DC-like kidney podocytes [20,21], or by interferon-γ in epithelial or endothelial cells [22]. In humans, this protein belongs to a family of six members (twelve members in mice) [23]. APOL3 appears to share the structure of other family members, as determined for APOL1 (Figure 1A). This structure is organized in a series of hydrophobic α-helices: an N-terminal four-helix bundle (helices 2–5) precedes a double-stranded helix potentially capable of transmembrane (TM) insertion, followed by another double-stranded helix involved in membrane interaction (membrane-addressing domain, or MAD), and a C-terminal leucine zipper helix (LZ) important for structural folding in the APOL1 case [24] (Figure 1A).

Like APOL1, APOL3 and mAPOL7C are induced by either TLR3 or interferon-γ signalling [21,27]. APOL1 and APOL3 share a similar peripheral location at Golgi and ER membranes, and they probably associate as they can weakly interact [21]. As a unique exception among all human or murine APOL family members, an APOL1 variant generated by differential mRNA splicing in human’s ancestor primates can be secreted [28]. Secreted APOL1 is mainly known for killing the African bloodstream parasite *Trypanosoma brucei* [28,29,30], as well as for kidney disease consequent to C-terminal mutations termed G1 and G2, which restore this killing activity on APOL1-resistant trypanosomes responsible for sleeping sickness [25,26,31]. Previously, it has been proposed that this disease results from two distinct effects of the C-terminal mutations: (i) interference of the intracellular APOL1 splice variants with APOL3 activity, resulting in actomyosin activity reorganization in podocytes [3,21]; (ii) interferon-induced increase in secreted APOL1 interaction with cholesterol (Figure 1B), resulting in activation of two cholesterol-controlled cationic channels at the podocyte surface and consecutive stress-linked cytotoxicity [26]. APOL3 is not secreted, but it participates in diverse processes related to intracellular membrane remodelling:

(1) Through hydrophobic interactions with PI4KB and PI4KB-controlling factors neuronal calcium sensor-1 (NCS1), calneuron-1 (CALN1) and ADP-ribosylation factor-1 (ARF1), APOL3 controls Golgi PI4P synthesis and PI4KB localization at the Golgi [3,21,24].
(2) Through helix interactions with vesicle-associated membrane protein 8 (VAMP8), APOL3 triggers the fusion between mitochondrial and endosomal membranes during mitophagy. This activity is promoted by cardiolipin [3].
(3) In epithelial cells, APOL3 is required for membrane melting of the cytosol-invasive Gram-negative bacterium *Salmonella tiphymurium* [22]. Such membrane fusion activity likely involves the capacity of APOL3 for pH-independent transmembrane insertion of a double-stranded helix hairpin [3], coupled to the selective APOL3 ability for cardiolipin solubilization [32], probably through interaction with a cardiolipin-binding motif in the second helix of the membrane-addressing domain [5]. For APOL1, such activities strictly depend on acidic conditions [3], as occurs in trypanosome endosomes where secreted APOL1 is taken up from the bloodstream [28,29,30], but it does not occur in APOL1-synthesizing cells, where none of the APOL1 splice variants reaches acidic compartments [26].
(4) In DCs, APOL3 or mAPOL7C is required for phagolysosomal membrane rupture linked to antigen cross-presentation [33].

In mice, mAPOL7C belongs to a subfamily of five members, which share high sequence similarity with APOL3 (around 40% identity and 55% similarity). Unlike APOL3, the mAPOL7 isoforms are characterized by the insertion of an acidic stretch separating helices 2–3 from helix 4 [24]. When disregarding this insert, mAPOL7C exhibits the highest similarity with APOL3 among all murine APOLs, suggesting a putative homology with APOL3 (44.4% identity; 60.9% similarity). However, the three-helix 2–4 bundle, containing the NCS1- and PI4KB-interacting helices 2 and 3 (or HC1) in APOL3 [3], is likely to be disrupted in mAPOL7C, possibly affecting these interactions.

Within the dynamin superfamily, GBP1 is a large GTPase belonging to a family of seven members. As occurs for APOL3 or APOL1, this protein is induced by either TLR3 or interferon-γ signalling [34]. Upon GTP binding, GBP1 assembles into higher-order structures coating pathogen-containing PVs or affecting cytosolic pathogens, leading to PV and/or bacterial membrane disruption that releases antigens from bacterial, viral, or protozoan pathogens, therefore inducing T cell activation and inflammation [34,35,36,37,38,39,40,41,42]. In *Salmonella*, GBP1 binds to the lipopolysaccharide coat, triggering danger pattern signalling and activating caspase 4 (or murine caspase 11) [43]. GBP1-induced disruption of pathogen-containing PV membranes could be related to its involvement in membrane repair, through C-terminal farnesyl anchor insertion in the membrane [37] and protein interaction with PIs [44]. However, APOL3/mAPOL7C was found to be a crucial component involved in megapore formation for antigen cross-presentation [33]. Thus, the membrane-disrupting activity of GBP1 may operate in synergy with APOL3/mAPOL7C. The proven association of both GBP1 and APOL3/mAPOL7C with phagolysosomal, vacuolar and intracellular bacterial membranes [22,33], together with the proven membrane-disrupting potential in both cases, suggests functional cooperation between these proteins.

GBP1 can also specifically bind to MCL1 (myeloid cell leukaemia 1), a key anti-apoptotic (pro-survival) member of the BCL2 (B-cell lymphoma 2) family [45]. In chronic myeloid leukaemia cells, GBP1 can neutralize MCL1 and drive BAK (Bcl-2 homologous antagonist/killer)-mediated apoptosis [45]. Moreover, GBP1-mediated caspase 4 activation [43,46,47] promotes BAX activity for apoptosis or pyroptosis [47,48,49]. Thus, GBP1 could be considered a pro-apoptotic factor, although this activity may differ depending on cell type or conditions. Along the same line, GBP1 can associate with the mitochondrion, promoting membrane fission [50,51]. Accordingly, the murine GBP1 homologue mGBP2 binds to cardiolipin, a prominent lipid of the mitochondrial membrane [35].

For both *Salmonella* lysis in epithelial cells and antigen cross-presentation in DCs, APOL3/mAPOL7C appears to work in synergy with GBP1 [22,33]. Moreover, the evidence that GBPs control the immune response against a large panel of intracellular pathogens, including bacteria, viruses, and protozoans [36,37,38,39,40,41,42,43,46,47], strongly suggests that such synergy generally applies to antigen cross-presentation.

## 3. Control of Myosins by Cardiolipin and Calmodulin-like Proteins

Calmodulin is an intracellular calcium receptor containing four double-stranded helix motifs undergoing conformational changes upon Ca^2+^ binding to the interhelical loop [52]. The calmodulin–Ca^2+^ complex binds to a specific hydrophobic region of myosin between the globular ATP-binding domain and the helical tail, which activates the motor force of myosins 5a and 6 [53,54]. This protein is representative of a large family including the two NM2A myosin light chains—ELC (essential light chain) and RLC (regulatory light chain)—which structurally and functionally replace calmodulin in the NM2A myosin [55].

Calmodulin-binding sequences, which can be predicted at http://calcium.uhnres.utoronto.ca/ctdb/ctdb/sequence.html (URL accessed in 1 August 2026), share several physicochemical similarities with sequences binding to anionic phospholipids such as PIs and cardiolipin. These sequences are rich in positively charged residues able to interact with either phosphate groups of phospholipids or calmodulin acidic residues. In addition, they often contain bulky hydrophobic residues allowing interaction with either the membrane lipid bilayer or calmodulin hydrophobic pockets [56,57,58,59,60,61,62,63]. Given such similarities, these sequences are used as regulatory control elements in several proteins [63,64,65,66,67]. In the case of different myosins, including NM2A, RLC phosphorylation induces its association with the heavy chain, and anionic phospholipids or cardiolipin can disrupt this interaction, regulating motor activity [54,67] (Figure 2). RLC phosphorylation can be performed by the myosin light chain kinase (MLCK), a Ca^2+^-calmodulin-dependent protein kinase possibly activated by TLR3-induced Ca^2+^ mobilization from internal ER stores [68,69].

Thus, infection-induced TLR3 signalling may trigger NM2A-driven membrane trafficking and/or contraction through alternative MYH9 interaction with either p-RLC or phospholipids. As detailed below, NM2A activities are likely to be important for the generation of phagolysosomal megapores allowing antigen cross-presentation.

## 4. Cardiolipin-Mediated BAX Activity in Diverse Membranes

Besides its interaction with the calmodulin-binding site of several myosins, cardiolipin is a key driver of mitochondrial megapore formation for apoptosis [19,70]. For this activity, cardiolipin interacts with BAX helix 6 (E131–L155) [70], which is the only predicted BAX calmodulin-binding sequence (I133–I152). Cardiolipin promotes the formation of BAX clusters at mitochondrial scission sites, near mitochondrion extremities in contact with the ER (MERCs) [71], where calcium fluxes from ER stores can trigger membrane fission promoted by PI4KB activation [9,72].

In the absence of cardiolipin, BAX megapore formation is improbable. Such is likely the case of the phagolysosomal membrane, unless exposure of cardiolipin occurs in this membrane during phagocytosis, due to membrane interaction with cardiolipin derived from pathogens or dead cells [73,74,75]. Consecutive damage-associated signalling could trigger APOL3, GBP1, and BAX recruitment to the phagolysosome, inducing megapore formation, similarly to what occurs in the mitochondrion [32,76]. Indeed, bacterial phagocytosis can induce apoptosis of macrophages [77], and cell death pathways can recruit BAX to lysosomal membranes, inducing lysosomal membrane permeabilization [78,79,80,81].

(Phago)lysosomal damage activates AMP-activated protein kinase [82], which can promote Golgi fragmentation induced by immunity-related GTPase M (IRGM) [83,84]. Similar to GBP1, IRGM is an interferon-inducible GTPase involved in pathogen control. IRGM prevents GBP1 from attacking intracellular membranes, while directing it toward PVs upon damage signalling [85,86,87]. Likewise, GBP1 participates in damage-induced Golgi fragmentation, as upon viral infection, IRGM binds to phosphorylated GBP1 to trigger Golgi stress [88,89,90]. In DCs or podocytes, Golgi fragmentation induces ARF1-mediated ATG9A vesicle traffic, releasing APOL3 and PI4KB from the Golgi, for PI4P-dependent fission of both phagolysosomal and mitochondrial membranes, coupling antigen cross-presentation with autophagy or apoptosis [2,3,5,91,92]. IRGM is required for this traffic, which also conditions STING degradation [93,94]. Similar to GBP1, APOL3 may be involved in Golgi remodelling, as APOL3 deletion or inhibition triggers Golgi shrinkage [21].

PI4KB-carrying ATG9A vesicle traffic should be distinguished from that of the endolysosomal PI4P kinase PIK42A, which, in contrast to PI4KB, is not known to interact with APOL3. Rather than membrane fission, PI4K2A promotes lysosomal membrane repair following Golgi-derived traffic of PI4K2A-carrying ATG9A vesicles to contact sites between the ER and the damaged lysosome, where PI4K2A-mediated PI4P synthesis allows the recruitment of lipid repair factors [95].

In conclusion, phagocytosis-linked cardiolipin exposure in the phagolysosomal membrane could trigger megapore formation in both the phagolysosome and the mitochondrion, and BAX could be involved in both cases.

## 5. APOL1 and APOL3 Association with the Three NM2A Subunits HC, ELC and RLC

The NM2A myosin drives the traffic of Golgi-derived ATG9A vesicles to MERCs for induction of autophagy and/or mitophagy [96,97,98] and participates in membrane fission and fusion events [99,100]. In APOL1 or APOL3 immunoprecipitates of human podocyte extracts, all three NM2A subunits (ELC, RLC and MYH9 HC) are present in almost stoichiometric amounts and represent the most abundant associated proteins [21]. Thus, NM2A is a prominent partner of APOL1 and APOL3, suggesting an important involvement of these APOLs in NM2A functions. Accordingly, APOL1 is required for ATG9A vesicle traffic induced by poly(I:C) in podocytes, and APOL3 is involved in both membrane fission and fusion processes [3,5,21].

Using bilayer interferometry for monitoring in vitro protein interaction, APOL3 exhibited high-affinity binding to the MYL12A RLC (K_D_ = 13 nM), which was favoured by either Mg^2+^ or Ca^2+^ (Figure 3A), consistent with the cation-binding properties of RLCs [101]. So far, APOL1 interaction with MYL12A was not measured.

In protein interaction assays in *Escherichia coli*, both APOL1 and APOL3 interacted with the MYL12A RLC (Figure 3B). APOL1 interaction with MYL12A was enhanced by truncation of the C-terminal leucine zipper helix (in APOL1Δ) (Figure 3B), in keeping with the increase in APOL1 hydrophobicity resulting from this truncation, which disrupts the APOL1-specific internal interaction between two hydrophobic clusters (HC1, HC2: see Figure 1A) [3,21,26].

In conclusion, the interaction of APOL1 or APOL3 with NM2A may occur through APOL binding to the calmodulin-like RLC. Such interaction may be involved in NM2A activation linked to phagolysosomal megapore formation for antigen cross-presentation.

## 6. Poly(I:C)-Induced Loss of p-RLC from APOL1 Immunoprecipitates

Incubation of human podocytes with the viral mimetic poly(I:C), which activates TLR3 signalling, strongly increases APOL1, APOL3, or mAPOL7 expression [20,21,27]. This treatment also enhances the association of the three NM2A subunits (ELC, RLC and MYH9 HC) with APOL1 [21], but it conversely triggers the selective disappearance of phosphorylated RLC (p-RLC) among NM2A subunits present in APOL1 immunoprecipitates (Figure 3C). The p-RLC loss was reduced in the APOL1Δ case, in keeping with the increased RLC binding to this variant (Figure 3B). Both the increase in NM2A subunit association and specific p-RLC dissociation highlight the role of APOL1 in poly(I:C)-triggered vesicular traffic driven by NM2A [3]. The loss of p-RLC from APOL1 immunoprecipitates is unlikely to result from dephosphorylation, because TLR3 signalling not only induces NM2A-mediated ATG9A vesicle traffic [3], but also promotes actomyosin cytoskeleton contraction, both activities depending on RLC phosphorylation [102,103,104,105]. Thus, poly(I:C)-induced p-RLC loss appears to result from selective dissociation.

I conclude that upon poly(I:C) treatment, p-RLC may switch from APOL1 to MYH9 association. Based on the structural similarities between the RLC and NCS1, which are both calmodulin-like proteins, the following considerations can be proposed:

(1) Whereas APOL3 interacts with Ca^2+^-NCS1, APOL1 does not [3,21]. Ca^2+^-NCS1 binds to APOL3 hydrophobic cluster 1 (HC1), which in APOL1 is blocked by interaction with a C-terminal HC2 [3,21] (Figure 1A). Increasing the accessibility of APOL1 HC1 through C-terminal deletion (in APOL1Δ) also increases RLC binding (Figure 3B), suggesting that RLC could bind to APOL1 HC1.
(2) In APOL3, Ca^2+^-NCS1 could prevent RLC binding to HC1.
(3) Poly(I:C) treatment triggers APOL1-mediated release of APOL3/PI4KB from the Golgi, following NM2A-driven ATG9A vesicle traffic to MERCs [3]. Thus, poly(I:C) may activate NM2A through p-RLC release from APOL1.
(4) p-RLC release from APOL1 could relate to increased APOL1 interaction with PI4P, because poly(I:C) promotes APOL1 traffic from PI4P-poor ER membranes to PI4P-rich Golgi vesicles [3,5,21], as also occurs for STING activation [4,5].

## 7. Preferential GBP1 Association with NM2A ELC and RLC Subunits

GBP1 strongly interacts with globular (G) actin, and weakly with filamentous (F) actin [106,107,108]. GBP1 interaction with G-actin, which prevents actin filament formation [107], underlies GBP1 control of infection by PV-enclosed and cytosolic pathogens, because GBP1-mediated impairment of actomyosin activity can limit the growth of many intracellular pathogens [109,110,111,112,113,114], except *Trypanosoma cruzi* [114]. Conversely, cross-presentation of dead-cell antigens involves increased NM2A activity on F-actin [115].

Like APOL1 and APOL3, GBP1 associates not only with PIs or cardiolipin [35,44], but also with the calmodulin-like NM2A subunits ELC and RLC [47]. Significantly, these subunits are prominent GBP1-associated components (respectively, ranked 5th and 8th among 1686 proteins, with the most abundant associated proteins being actin and caspase 4) [47]. Based on the search for calmodulin-binding sequences, the putative PIs-/cardiolipin-binding sites of APOL1, APOL3 and GBP1 are defined in Figure 4 (box), together with those of BAX and the BAX-activating caspase 4 (which is inhibited by cardiolipin [116]). In GBP1, this site precisely corresponds to helix 10 (G432–E449), located in the middle domain core bundle, which contributes to stabilizing the elongated shape of the C-terminal domain of the protein, supposed to be membrane-proximal owing to the presence of polybasic motifs favouring interaction with PI4P [35,44,117]. Upon GTP binding, helix 10 exhibits one of the highest flexibility and dynamic changes of the entire protein [35], and consistently, under these conditions, this helix could possibly interact with intracellular membranes. Interestingly, despite the important abundance of both ELC and RLC in the GBP1 immunoprecipitates, the NM2A heavy chain (MYH9) is only poorly represented (ranked 340th) [47]. This observation contrasts sharply with the APOL1 case, where all three NM2A subunits were detected in near-stoichiometric amounts, with MYH9 ranked 1st among associated proteins [21]. Thus, GBP1 preferentially associates with NM2A regulatory factors, possibly released from interaction with MYH9. This interaction is likely to involve helix 10 and to be regulated by cardiolipin.

In conclusion, like APOL1 or APOL3, GBP1 could share reversible interaction with NM2A light chains to control myosin activity.

## 8. Synergistic Activity of APOL3 and GBP1 in Membrane Megapore Formation: A Model

In the schematic model presented in Figure 4, APOL3 and GBP1 contribute to the formation of membrane megapores underlying antigen cross-presentation. The expression of both proteins is induced by either TLR3 or interferon-γ signalling, conditions also promoting GTP binding to the ARF1, IRGM, and GBP1 GTPases [83,84,85,87]. Whereas ARF1 activation induces Golgi-derived ATG9A vesicle trafficking for the transport of PI4P kinases to various endomembranes [2,3,72,89,90], GTP binding to GBP1 induces a major restructuring of this protein into higher-order complexes, disorganizing the membrane coat of both PVs and intracellular pathogens [34,35,36,37,38,39,112,113]. In each case, GBP1 activity is expected to facilitate membrane access by external proteins such as APOL3 (or mAPOL7C). In contrast to APOL3, APOL1 was not identified as a key player in antigen cross-presentation [33]. This observation is consistent with the inability of intracellular APOL1 to generate membrane pores at cytosolic pH [26,119]. In podocytes, APOL1 was rather involved in intracellular traffic of Golgi-derived ATG9A vesicles [3]. Thus, the APOL3 involvement in cross-presentation is distinct from ATG9A vesicle traffic.

More pertinently, APOL3/mAPOL7C can perform deep membrane remodelling through pH-independent transmembrane insertion of a flexible double-stranded helix favouring membrane melting [3,5,24,119], linked to specific cardiolipin solubilization involving a calmodulin-binding motif in the second helix of the membrane-addressing domain [5,32]. As demonstrated in direct membrane fusion assays in vitro [3], cardiolipin promotes APOL3 membrane fusion activity, and mutating the APOL3 membrane-addressing domain totally prevents membrane fusion. Regarding the PIs-/cardiolipin-binding potential of GBP1 [35], it may also contribute to membrane disruption, although sequestering NM2A RLC/ELC can also be envisaged.

According to the APOL3 membrane fusion activity exerted between mitophagosomal and endolysosomal membranes [3], a role for endosomal VAMP8, the APOL3 partner for membrane fusion, can be expected. Consistently, VAMP8 is present at the phagolysosomal membrane during phagocytosis, where it is known to be crucially involved in antigen cross-presentation [120]. Currently, the role attributed to VAMP8 in antigen cross-presentation is the recruitment of NADPH oxidase 2 (NOX2) to the phagolysosome, which triggers lipid peroxidation, a membrane-disrupting chain reaction expected to result in antigen leakage from the vacuole [121,122]. However, such a non-specific destructive process is unlikely to induce the formation of membrane megapores that would still preserve the overall phagolysosomal structure. Moreover, APOL3 (or mAPOL7C), rather than NOX2, was identified as the key factor for phagosomal megapore formation [33]. Therefore, VAMP8’s role in antigen cross-presentation may involve APOL3. Accordingly, initiation of phagosomal megapore formation may result from APOL3-mediated cardiolipin solubilization occurring during membrane fusion between cardiolipin-exposing phagosomal membranes and VAMP8-exposing endolysosomal membranes for phagolysosome formation [5,72]. In support of this model, GBP1 is involved in membrane remodelling of both phagosomal and endolysosomal membranes [44].

After the generation of discrete pores at the PIs-/cardiolipin-exposing sites, phagolysosomal pore expansion into megapores could require APOL3- and GBP1-dependent activation of both NM2A and BAX. On one hand, depending on local Ca^2+^ concentration influenced by TLR3 signalling [68,69,123], the NM2A p-RLC could be released from interaction with APOL1/APOL3/mAPOL7C for association with MYH9 and consecutive induction of the NM2A motor activity on F-actin. Similarly, the RLC/ELC association with GBP1 [47] could be influenced by interferon signalling, contributing to NM2A activation. Such activation could be required for membrane stretching linked to expansion and maintenance of the open state of the pore, as occurs for pore expansion and stabilization during exocytosis [124,125,126], a process where, significantly, APOL2 or mAPOL8 appear to be crucially involved [5,127]. Specifically, NM2A could counteract membrane tension to prevent premature closure of the megapore [128]. In the case of apoptotic mitochondrial megapores, such collapse prevention is strengthened by the building of a rigid inner wall consisting of cardiolipin-interacting BAX/BAK oligomers [129]. Consistently, both APOL3/mAPOL7 and GBP1 can interact with anti-apoptotic BCL2-like proteins [20,45] to promote BAX/BAK involvement in megapore stabilization. Whereas this clearly occurs for apoptotic megapores of the outer mitochondrial membrane, the involvement of BAX/BAK in the building of phagolysosomal megapores is not known. However, BAX can be recruited to endolysosomal membranes [78,79,80,81], and inhibition or deletion of anti-apoptotic BCL2 increases antigen presentation [130], suggesting that activated BAX could be involved not only in apoptosis, but also in phagolysosomal megapore formation.

Alternatively, or additionally, APOL3/mAPOL7C could replace BAX/BAK to form megapores in phagolysosomal membranes:

(1) In both murine DCs and human podocytes, mAPOL7 and APOL3 exhibit BAX-related activity, since they are required for apoptosis induced by poly(I:C) [20,21]. This could result from their ability to interact with the B-cell lymphoma-extra large (Bcl-xL) anti-apoptotic protein [20], but a direct role in megapore formation cannot be ruled out.
(2) Like BAX, different APOLs, including APOL3, contain a helix resembling the BCL2-homology motif 3 (BH3) [131] (Figure 4, box). In BAX, this helix is essential for apoptotic pore formation [132]. In mAPOL7C and APOL1, the corresponding helix is involved in mitochondrial membrane permeabilization in DCs and in trypanosomes, respectively [20,30]. Accordingly, APOL3 can also induce trypanosome mitochondrial membrane permeabilization like APOL1 [119].
(3) The flexibility of the APOL3/mAPOL7C transmembrane helices (high proportion of β-branched [Ile/Val] and helix-breaking [Gly/Pro/Ser/Thr] amino acids) evokes that of the BAX transmembrane amphipathic helix 9 (Figure 4, box). BAX helix 9 is crucially involved in megapore wall organization, through both its amphipathic character and the presence of a characteristic GxxxA motif [132,133,134], known, like GxxxG, to promote oligomeric association between transmembrane helices [134,135,136,137,138,139,140]. Significantly, the APOLs transmembrane helices not only contain GxxxA and GxxxG motifs, but also other small-residue packing motifs (G/A/S/TxxxG/A/S/T) that can induce tight association between protein helices in vitro [118]. In fact, almost the totality of the APOLs’ transmembrane helices is made of such motifs (Figure 4, box). Thus, these helices are predicted to potentially organize megapore-forming oligomeric structures, as occurs for BAX/BAK complexes.
(4) As mentioned above, the oligomerization of mitochondrial BAX strongly depends on cardiolipin, whose interaction with helix 6 ensures BAX clamping on the membrane [133]. Cardiolipin is enriched in the mitochondrial inner membrane and can be exposed at the outer mitochondrial membrane, but it is probably largely absent from phagosomal membranes. Thus, BAX may preferentially work in mitochondrial membranes.
(5) Probably owing to a MAD-mediated membrane-clamping effect analogous to that of BAX helix 6, mitophagosome membrane fusion by APOL3 is promoted by cardiolipin [3], and APOL3-dependent megapore formation in the mitochondrial inner membrane also crucially involves cardiolipin [32]. However, the lipid dependence of APOL3 oligomerization is unknown, and PIs of the phagolysosomal membrane, like PI4P, which can strongly bind to APOL3 [21], could possibly also exert this activity. Thus, APOL3/mAPOL7C could be involved in megapore formation in phagolysosomal membranes.

In conclusion, following GBP1-mediated access to phagolysosomal and/or pathogen membranes, APOL3/mAPOL7C could act in initiation, expansion, and stabilization of membrane megapores. This would result from a combination of intrinsic membrane remodelling properties, oligomerization induced by tight interactions between transmembrane α-helices, and promotion of actomyosin activity.

## 9. Discussion

Despite their extensive sequence and structure differences, APOL3 and GBP1 exhibit functional similarities. In addition to a common induction by interferon, these proteins share a common involvement in phagolysosome membrane permeabilization and consecutive activation of the immune system, together with common pro-apoptotic activity and similar prominent association with regulatory components of the NM2A myosin. Thus, upon infection, these proteins appear to cooperate to trigger megapore formation in the phagolysosomal membrane. Whereas GBP1 associates with peripheral membrane components and/or the cytosolic side of intracellular membranes through a C-terminal lipid anchor, APOL3 can perform pH-independent transmembrane insertion and cardiolipin solubilization, suggesting that GBP1 helps pore formation by APOL3. When inserted in the membrane, the strong oligomerization potential of the APOL3 transmembrane spans evokes BAX/BAK ability to generate oligomeric megapore inner walls, and the regulatory potential of both APOL3 and GBP1 on NM2A motor activity suggests NM2A involvement in membrane stretching linked to expansion and stabilized opening of the megapore.

Two additional factors, namely mitochondrial complex I (CI) [141] and perforin-2 (P2) [142], were recently linked to antigen cross-presentation. Since CI activity promotes ATP synthesis by contributing to the proton-motive force across the inner mitochondrial membrane, CI could be required for ATP-dependent NM2A contraction activity underlying membrane megapore opening. Regarding P2, oligomeric pore formation at the DC plasma membrane promotes macropinocytic antigen uptake, while within endosomes, P2 allows acidity reduction to prevent excessive antigen degradation and facilitate cross-presentation [142]. Thus, contrasting with APOL3/mAPOL7C, P2 does not seem (so far) to be involved in phagolysosomal megapore formation.

## 10. Conclusions

GBP family proteins, particularly GBP1, have clearly been demonstrated as important components of autonomous cellular immunity, through their ability to induce antigen release from quite diverse intravacuolar or cytoplasmic pathogens and subsequent T cell response activation. Likewise, human APOL3 (or murine mAPOL7C) was recently found to be a key factor for antigen cross-presentation and T cell activation resulting from antigen release from phagolysosomal or intracellular bacterial membranes. Given their common localisation and function, APOL3 and GBP1 could hypothetically work in synergy to induce intracellular membrane permeabilization leading to antigen cross-presentation. The aim of this perspective was to evaluate this proposal, considering the structural and functional properties of each protein. A model is presented, where GBP1 and APOL3, respectively, contribute to membrane disorganization and membrane permeabilization, while both induce BAX and NM2A myosin activation for megapore expansion and maintenance in an open state.

Although supported by various publications in the field, the model presented here is obviously incomplete and remains largely speculative. Its main objective is to encourage research along the presented lines. Moreover, reports of differing APOL3 and GBP1 activities in different cell types or conditions evidently indicate that these proteins are subjected to complex controls, and the existence of several isoforms in both cases further adds to this complexity.

## Figures and Tables

**Figure 1:**
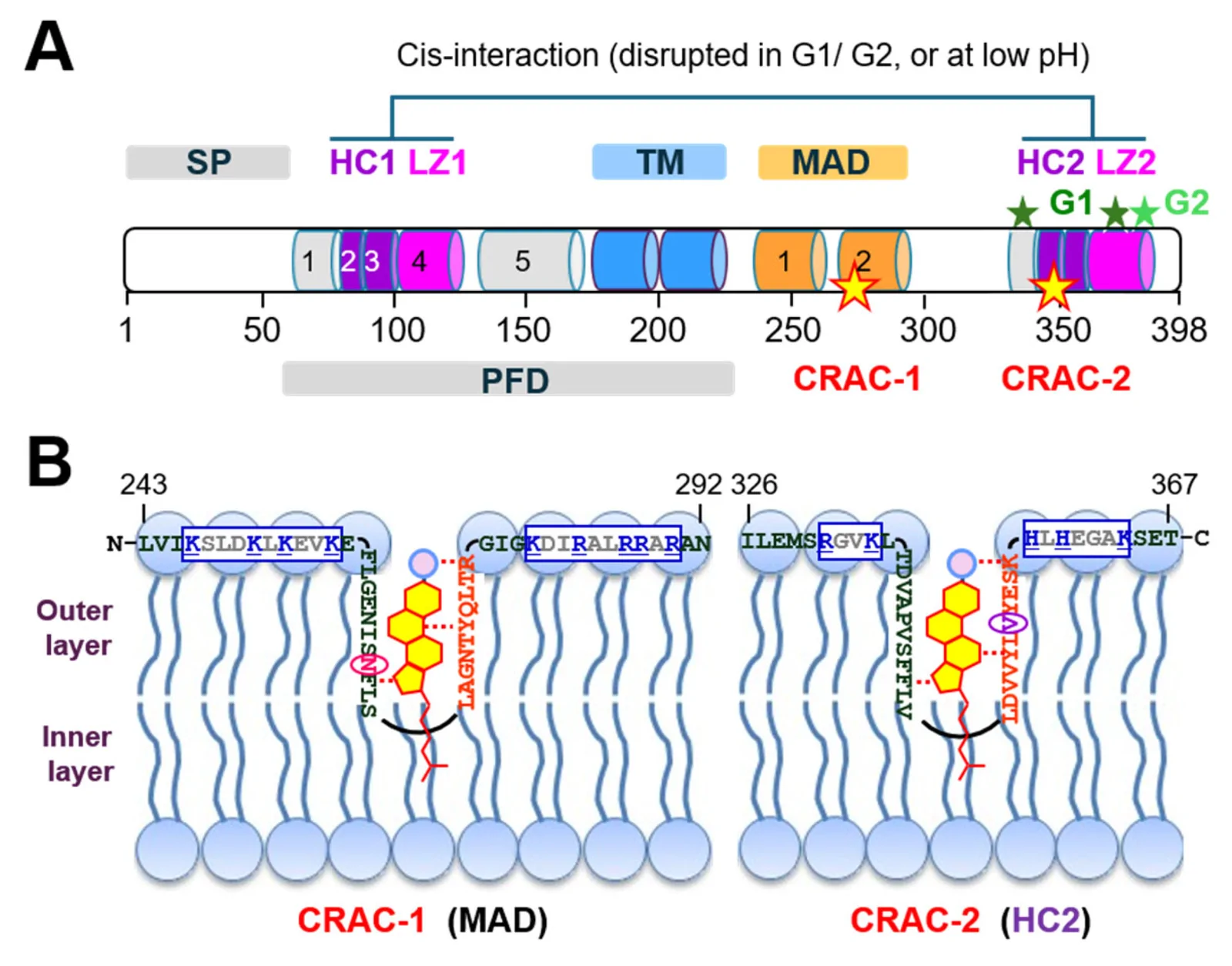
**APOL1 structure and cholesterol binding.** (**A**) Schematic structure of the secreted APOL1 splice variant. The coloured cylinders represent different α-helices (SP: signal peptide; HC: hydrophobic cluster; LZ: leucine zipper; TM: transmembrane span; MAD: membrane-addressing domain; PFD: pore-forming domain; CRAC: cholesterol recognition amino acid consensus). The kidney disease-linked G1 and G2 mutations [25] are indicated by dark and light green stars, respectively, and the CRAC motifs are indicated by yellow stars. (**B**) Models of APOL1 CRAC insertion in the plasma membrane. The CRAC sequences, coloured red, are flanked by putative phospholipid-binding sequences framed in blue, with positively charged residues in blue. Mutation of N264 (encircled and coloured pink) into K264 reduces APOL1 G1/G2 cytotoxicity, possibly owing to interference with cholesterol binding [26]. V353 (encircled and coloured violet) is the C-terminal residue of APOL1Δ (see text). Cholesterol is represented in yellow, with a red contour.

**Figure 2:**
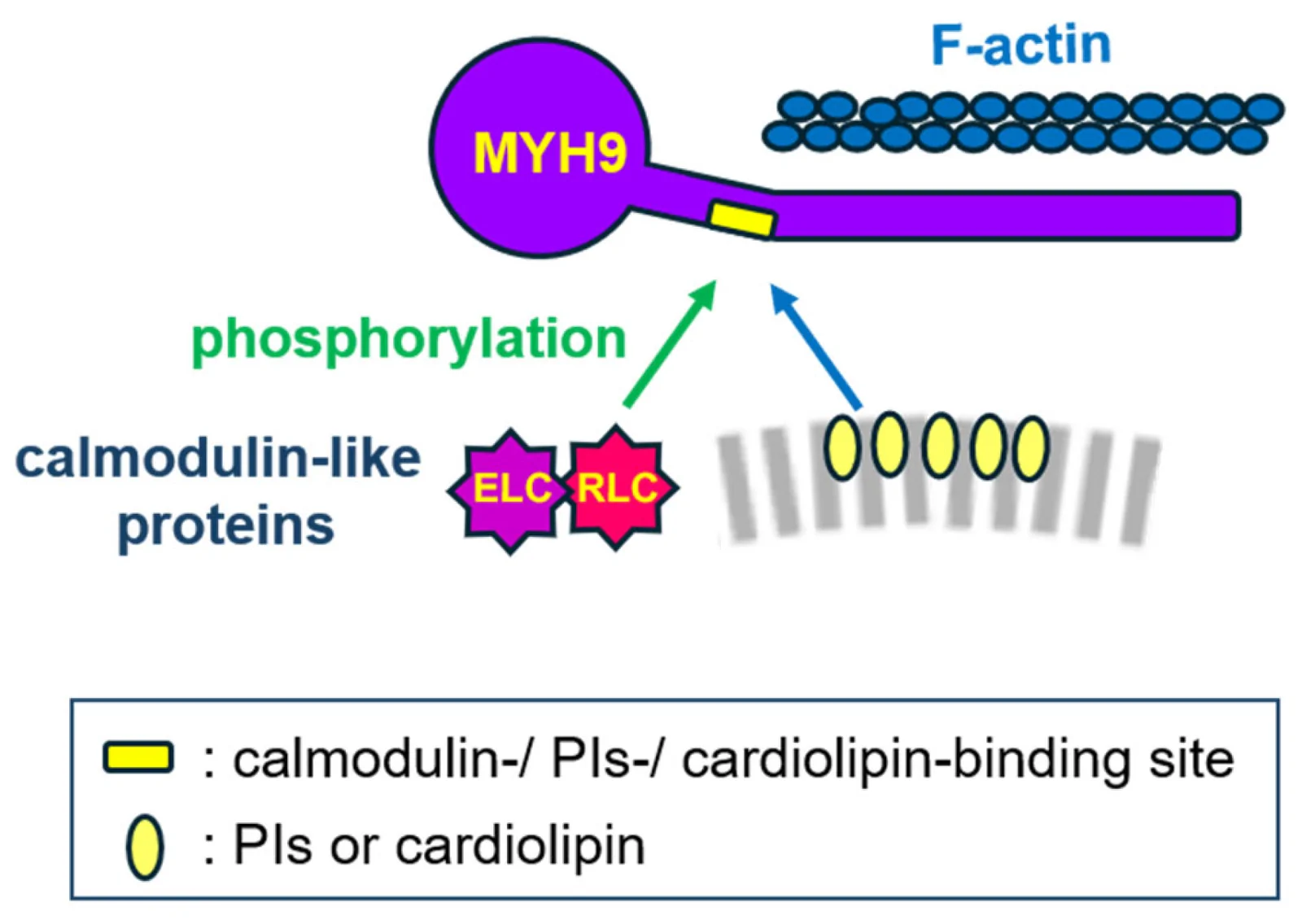
**The dual binding activity of the NM2A heavy chain (HC) MYH9.** The PIs-/cardiolipin-binding site located at the neck of MYH9 can interact either with phosphorylated RLC (p-RLC) or with membrane anionic phospholipids, influencing NM2A activities. Whereas the green arrow refers to proven activation of NM2A following p-RLC interaction, the blue arrow means that regarding NM2A, the effect of anionic phospholipids binding is not known.

**Figure 3:**
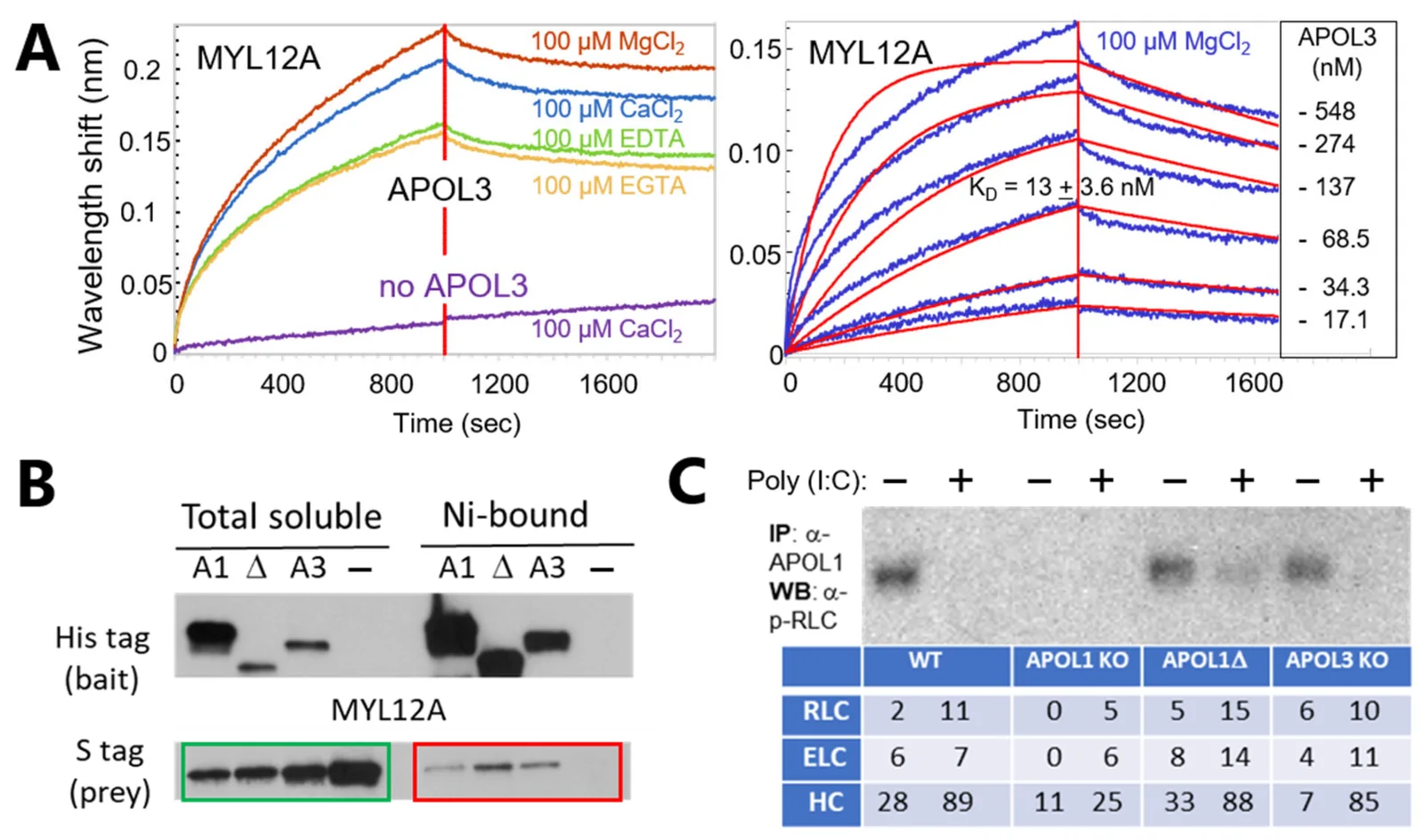
**Binding of APOL3 and APOL1 to the NM2A RLC.** (**A**) Bilayer interferometry measurement of the interaction between immobilized RLC MYL12A and soluble APOL3. The dissociation constant (K_D_) was determined from three experiments (**right panel**). (**B**) APOL1, APOL1Δ, or APOL3 interaction with MYL12A in *Escherichia coli*, following their co-expression in the pDUET plasmid (Novagen). The protein partners contain different C-terminal tags: the APOLs’ 6-histidine tag allows protein binding to a nickel matrix, whereas the MYL12A S-tag does not. Thus, matrix-associated S-tagged MYL12A is considered bound to APOL. The bands in the green frame reveal MYL12A proteins engaged in the interaction, whereas those in the red frame reveal MYL12A complexed with APOLs. Ni: nickel; APOL1Δ: APOL1 truncated at V353. (**C**) Western blotting evidence of poly(I:C)-induced dissociation of p-RLC from APOL1. In APOL1 immunoprecipitates of podocytes expressing wild-type (WT) or edited APOL1/APOL3, the specific anti-phosphorylated MYL12A (p-RLC) antibodies (Abcam) no longer reveal RLC following cellular treatment with the viral mimetic poly(I:C). The table shows the numbers of NM2A polypeptides detected by mass spectrometry analysis of each immunoprecipitate. Residual polypeptides in APOL1 KO immunoprecipitates may be due to co-precipitation of APOL2, which exhibits 61.4% identity/74.6% similarity with APOL1. IP: immunoprecipitation; WB: Western blotting; HC: NM2A heavy chain (MYH9); APOL1Δ: APOL1 truncated at V353. All methods are described in [21]. The figure is from the corresponding author’s laboratory, and there are no copyright concerns.

**Figure 4:**
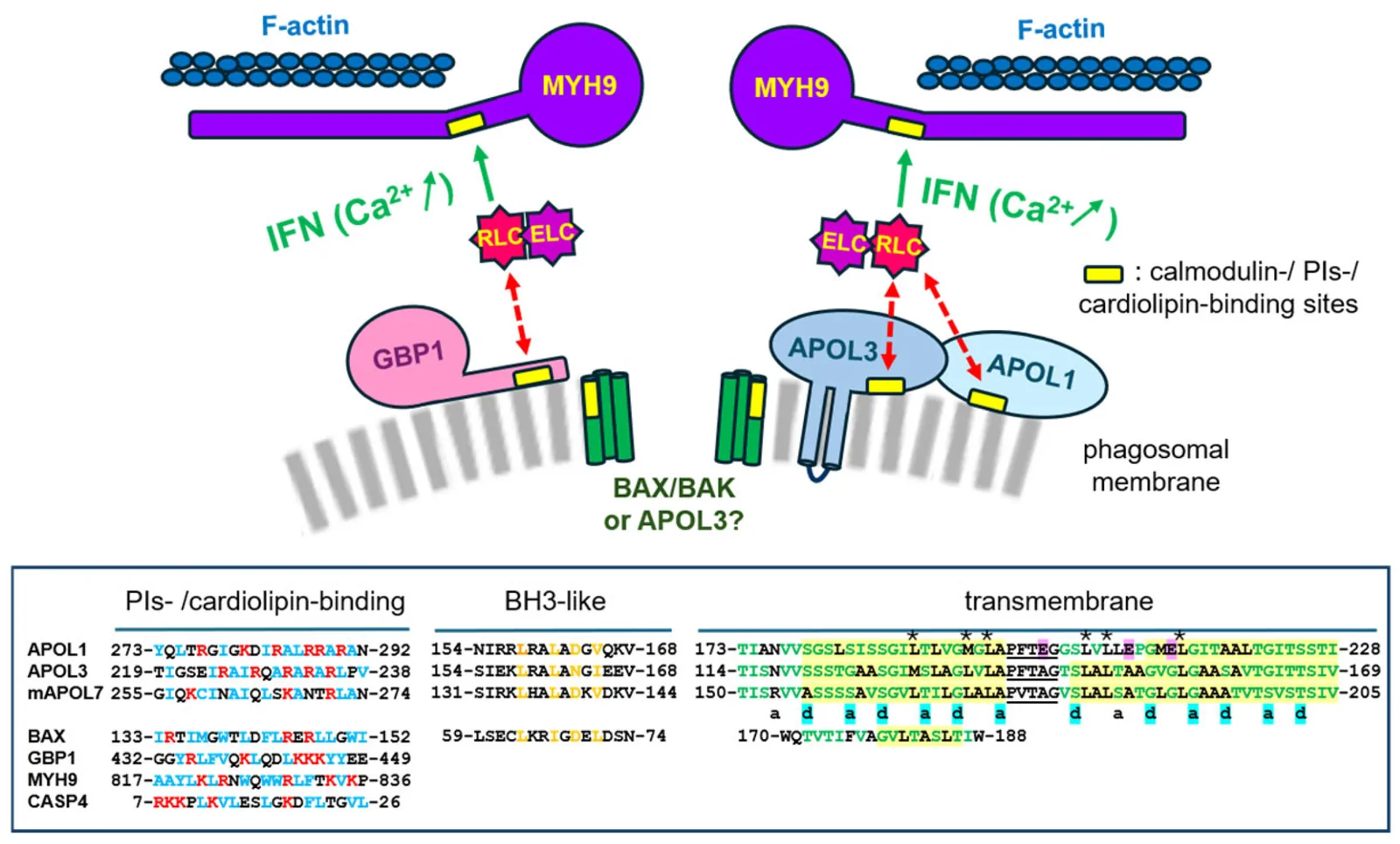
**Synergistic activity between GBP1 and APOL1/3.** Both GBP1 and APOL1/3 can interact with PIs or cardiolipin, NM2A light chains (ELC/RLC), and anti-apoptotic BCL2 family members. TLR3 or interferon-γ signalling induces processes affecting these interactions: (i) strong increase in the expression of both APOL1/3 and GBP1; (ii) GBP1 phosphorylation, inducing major conformational changes that disrupt the phagosomal membrane structure for improved access of the pore-forming protein APOL3; (iii) p-RLC release from APOL1, possibly activating NM2A and consecutive actomyosin contraction for pore enlargement and stabilization into an open state; (iv) GBP1 and/or APOL1/3 interaction with BCL2 family members, activating BAX for the building of megapore-coating oligomeric structures. APOL1/3 and BAX α-helices share functional similarities, possibly explaining why APOL1/3 exert BAX-like activities [20,21,72]: (i) putative PIs-/cardiolipin-binding sites, as determined by calmodulin-binding prediction (http://calcium.uhnres.utoronto.ca/ctdb/ctdb/sequence.html) (URL accessed in 1 August 2026) (positively charged and hydrophobic residues are, respectively, coloured red and blue); (ii) BCL2-homology motif 3 (BH3)-like helices, found to be functional in murine APOL3 (mAPOL7C) and APOL1 [20,30] (key BH3 residues are coloured orange); (iii) transmembrane helices, sharing not only a high content in destabilizing amino acids (coloured green) but also small-residue packing motifs (G/A/S/TxxxG/A/S/T; highlighted in yellow), which can experimentally promote helix clustering [118]. The “a” and “d” characters designate the 1st and 4th residues of helix heptad repeats, located on the same helix side. These residues are highlighted in blue if they are small in all three APOLs. Residues highlighted in pink are responsible for APOL1’s absolute dependency on acidic pH for transmembrane insertion and membrane fusion [3,119], such that only APOL3 exhibits transmembrane helices. IFN: interferon; underlined sequences: interhelical loops; asterisks: conserved (structuring?) leucine pattern. While p-RLC binding is known to activate NM2A (green arrows), the reversible p-RLC sequestration by APOLs (and GBP1?) is expected to exert an opposite effect (dotted red arrows).

## Data Availability

All data supporting the findings of this publication are available within this article.

## References

[1] Bancilhon M.D., Mesmin B. (2026). Reciprocal control of viral infection and phosphoinositide dynamics. FEBS Lett..

[2] Judith D., Jefferies H.B., Boeing S., Frith D., Snijders A.P., Tooze S.A. (2019). ATG9A shapes the forming autophagosome through arfaptin 2 and phosphatidylinositol 4-kinase IIIβ. J. Cell Biol..

[3] Lecordier L., Heo P., Graversen J.H., Hennig D., Skytthe M.K., d’ELzius A.C., Pincet F., Pérez-Morga D., Pays E. (2023). Apolipoproteins L1 and L3 control mitochondrial membrane dynamics. Cell Rep..

[4] Fang R., Jiang Q., Jia X., Jiang Z. (2023). ARMH3-mediated recruitment of PI4KB directs Golgi-to-endosome trafficking and activation of the antiviral effector STING. Immunity.

[5] Pays E. (2026). Apolipoproteins L involvement in immunity. J. Hum. Immun..

[6] Dippold H.C., Ng M.M., Farber-Katz S.E., Lee S.-K., Kerr M.L., Peterman M.C., Sim R., Wiharto P.A., Galbraith K.A., Madhavarapu S. (2009). GOLPH3 bridges phosphatidylinositol-4-phosphate and actomyosin to stretch and shape the Golgi to promote budding. Cell.

[7] Kuna R.S., Field S.J. (2019). GOLPH3: A Golgi phosphatidylinositol(4)phosphate effector that directs vesicle trafficking and drives cancer. J. Lipid Res..

[8] Waugh M.G. (2019). The Great Escape: How phosphatidylinositol 4-kinases and PI4P promote vesicle exit from the Golgi (and drive cancer). Biochem. J..

[9] Nagashima S., Tábara L.-C., Tilokani L., Paupe V., Anand H., Pogson J.H., Zunino R., McBride H.M., Prudent J. (2020). Golgi-derived PI(4)P-containing vesicles drive late steps of mitochondrial division. Science.

[10] De Tito S., Hervás J.H., van Vliet A.R., Tooze S.A. (2020). The Golgi as an assembly line to the autophagosome. Trends Biochem. Sci..

[11] Duan X., Wei Y., Zhang M., Zhang W., Huang Y., Zhang Y.-H. (2023). PI4P-containing vesicles from Golgi contribute to mitochondrial division by coordinating with polymerized actin. Int. J. Mol. Sci..

[12] Guo X., Wei L., Kundu N., Shin W., Chan C.Y., Han S., Mohseni A., Molakarimi M., Sun M., Wu X.-S. (2025). A phosphoinositide switch from PI(4,5)P_2_ to PI4P triggers endocytosis by inducing dynamin-mediated fission in secretory cells. Sci. Adv..

[13] Li K., Underhill D.M. (2025). Phagocytosis: A process that shapes immune responses to engulfed meals. Nat. Rev. Immunol..

[14] Zhang G., Liu J., Li W., Wang B., Feng H., Cui T., Pan T., Chen D., Xiong Y. (2026). The signaling and regulatory functions of dying cells and cell corpses. Commun. Biol..

[15] Jeschke A., Zehethofer N., Lindner B., Krupp J., Schwudke D., Haneburger I., Jovic M., Backer J.M., Balla T., Hilbi H. (2015). Phosphatidylinositol 4-phosphate and phosphatidylinositol 3-phosphate regulate phagolysosome biogenesis. Proc. Natl. Acad. Sci. USA.

[16] Kumar Y., Valdivia R.H. (2009). Leading a sheltered life: Intracellular pathogens and maintenance of vacuolar compartments. Cell Host Microbe.

[17] Clough B., Frickel E.-M. (2017). The Toxoplasma Parasitophorous Vacuole: An Evolving Host–Parasite Frontier. Trends Parasitol..

[18] Ohara R.A., Murphy K.M. (2023). Recent progress in type 1 classical dendritic cell cross-presentation-cytosolic, vacuolar, or both?. Curr. Opin. Immunol..

[19] Gonzalvez F., Gottlieb E. (2007). Cardiolipin: Setting the beat of apoptosis. Apoptosis.

[20] Uzureau S., Coquerelle C., Vermeiren C., Uzureau P., Van Acker A., Pilotte L., Monteyne D., Acolty V., Vanhollebeke B., Eynde B.V.D. (2016). Apolipoproteins L control cell death triggered by TLR3/TRIF signaling in dendritic cells. Eur. J. Immunol..

[21] Uzureau S., Lecordier L., Uzureau P., Hennig D., Graversen J.H., Homblé F., Mfutu P.E., Arcolino F.O., Ramos A.R., La Rovere R.M. (2020). APOL1 C-terminal variants may trigger kidney disease through interference with APOL3 control of actomyosin. Cell Rep..

[22] Gaudet R.G., Zhu S., Halder A., Kim B.-H., Bradfield C.J., Huang S., Xu D., Mamiñska A., Nguyen T.N., Lazarou M. (2021). A human apolipoprotein L with detergent-like activity kills intracellular pathogens. Science.

[23] Page N.M., Butlin D.J., Lomthaisong K., Lowry P.J. (2001). The human apolipoprotein L gene cluster: Identification, classification, and sites of distribution. Genomics.

[24] Pays E. (2024). Apolipoprotein-L functions in membrane remodeling. Cells.

[25] Genovese G., Friedman D.J., Ross M.D., Lecordier L., Uzureau P., Freedman B.I., Bowden D.W., Langefeld C.D., Oleksyk T.K., Knob A.L.U. (2010). Association of trypanolytic ApoL1 variants with kidney disease in African Americans. Science.

[26] Pays E. (2024). The two levels of podocyte dysfunctions induced by apolipoprotein L1 risk variants. Kidney Dial..

[27] Nichols B., Jog P., Lee J.H., Blackler D., Wilmot M., D’Agati V., Markowitz G., Kopp J.B., Alper S.L., Pollak M.R. (2015). Innate immunity pathways regulate the nephropathy gene Apolipoprotein L1. Kidney Int..

[28] Pays E., Vanhollebeke B., Uzureau P., Lecordier L., Pérez-Morga D. (2014). The molecular arms race between African trypanosomes and humans. Nat. Rev. Microbiol..

[29] Vanhamme L., Paturiaux-Hanocq F., Poelvoorde P., Nolan D.P., Lins L., Van Den Abbeele J., Pays A., Tebabi P., Van Xong H., Jacquet A. (2003). Apolipoprotein L-I is the trypanosome lytic factor of human serum. Nature.

[30] Vanwalleghem G., Fontaine F., Lecordier L., Tebabi P., Klewe K., Nolan D.P., Yamaryo-Botté Y., Botté C., Kremer A., Burkard G.S. (2015). Coupling of lysosomal and mitochondrial membrane permeabilization in trypanolysis by APOL1. Nat. Commun..

[31] Daneshpajouhnejad P., Kopp J.B., Winkler C.A., Rosenberg A.Z. (2022). The evolving story of apolipoprotein L1 nephropathy: The end of the beginning. Nat. Rev. Nephrol..

[32] Ritacco D.A., Shahnawaz H., Oduguwa A., Hawk J., Vizcaino B., Farber D.L., Gaudet R.G. (2026). The human antibacterial factor APOL3 couples lysosomal damage to mitochondrial DNA efflux and type I IFN induction. Mol. Cell.

[33] Gonzales G.A., Huang S., Wilkinson L., Nguyen J.A., Sikdar S., Piot C., Naumenko V., Rajwani J., Wood C.M., Dinh I. (2024). The pore-forming apolipoprotein APOL7C drives phagosomal rupture and antigen cross-presentation by dendritic cells. Sci. Immunol..

[34] Kutsch M., Coers J. (2021). Human guanylate binding proteins: Nanomachines orchestrating host defense. FEBS J..

[35] Loschwitz J., Steffens N., Wang X., Schäffler M., Pfeffer K., Degrandi D., Strodel B. (2023). Domain motions, dimerization, and membrane interactions of the murine guanylate binding protein 2. Sci. Rep..

[36] Zhu S., Bradfield C.J., Maminska A., Park E.-S., Kim B.-H., Kumar P., Huang S., Kim M., Zhang Y., Bewersdorf J. (2024). Native architecture of a human GBP1 defense complex for cell-autonomous immunity to infection. Science.

[37] Weismehl M., Chu X., Kutsch M., Lauterjung P., Herrmann C., Kudryashev M., Daumke O. (2024). Structural insights into the activation mechanism of antimicrobial GBP1. EMBO J..

[38] Kuhm T., Taisne C., Pinto C.d.A., Gross L., Giannopoulou E.A., Huber S.T., Pardon E., Steyaert J., Tans S.J., Jakobi A.J. (2024). Structural basis of antimicrobial membrane coat assembly by human GBP1. Nat. Struct. Mol. Biol..

[39] Kutsch M., Herrmann C. (2026). Glue it together: Self-assembly of the antibacterial GTPase GBP1. Biol. Chem..

[40] Pilla-Moffett D., Barber M.F., Taylor G.A., Coers J. (2016). Interferon-inducible GTPases in host resistance, inflammation and disease. J. Mol. Biol..

[41] Glitscher M., Himmelsbach K., Woytinek K., Schollmeier A., Johne R., Praefcke G.J.K., Hildt E. (2021). Identification of the interferon-inducible GTPase GBP1 as a major restriction factor for hepatitis E virus. J. Virol..

[42] Farrukee R., Mordant F., Mackenzie-Kludas C., Mesner D., Yamomoto M., Jolly C., Brooks A.G., Subbarao K., Londrigan S.L., Reading P.C. (2025). Human guanylate-binding protein (GBP) 1 inhibits replication of severe acute respiratory syndrome coronavirus 2. J. Virol..

[43] Santos J.C., Boucher D., Schneider L.K., Demarco B., Dilucca M., Shkarina K., Heilig R., Chen K.W., Lim R.Y.H., Broz P. (2020). Human GBP1 binds LPS to initiate assembly of a caspase-4 activating platform on cytosolic bacteria. Nat. Commun..

[44] Buijze H., Brinkmann V., Hurwitz R., Dorhoi A., Kaufmann S.H.E., Pei G. (2023). Human GBP1 is involved in the repair of damaged phagosomes/endolysosomes. Int. J. Mol. Sci..

[45] Luo Y., Jin H., Kim J.H., Bae J. (2021). Guanylate-binding proteins induce apoptosis of leukemia cells by regulating MCL-1 and BAK. Oncogenesis.

[46] Wandel M.P., Kim B.-H., Park E.-S., Boyle K.B., Nayak K., Lagrange B., Herod A., Henry T., Zilbauer M., Rohde J. (2020). Guanylate-binding proteins convert cytosolic bacteria into caspase-4 signaling platforms. Nat. Immunol..

[47] Fisch D., Clough B., Domart M.-C., Encheva V., Bando H., Snijders A.P., Collinson L.M., Yamamoto M., Shenoy A.R., Frickel E.-M. (2020). Human GBP1 differentially targets salmonella and toxoplasma to license recognition of microbial ligands and caspase-mediated death. Cell Rep..

[48] Abu Khweek A., Amer A.O. (2020). Pyroptotic and non-pyroptotic effector functions of caspase-11. Immunol. Rev..

[49] Zhang H., Zhang J., Luan S., Liu Z., Li X., Liu B., Yuan Y. (2023). Unraveling the complexity of regulated cell death in esophageal cancer: From underlying mechanisms to targeted therapeutics. Int. J. Biol. Sci..

[50] Qiu X., Guo H., Yang J., Ji Y., Wu C.-S., Chen X. (2018). Down-regulation of guanylate binding protein 1 causes mitochondrial dysfunction and cellular senescence in macrophages. Sci. Rep..

[51] Kalb R.C., Nyabuto G.O., Morran M.P., Maity S., Justinger J.S., Nestor-Kalinoski A.L., Vestal D.J. (2024). The large GTPase guanylate-binding protein-1 (GBP-1) promotes mitochondrial fission in glioblastoma. Int. J. Mol. Sci..

[52] Zhang M., Abrams C., Wang L., Gizzi A., He L., Lin R., Chen Y., Loll P.J., Pascal J.M., Zhang J.-F. (2012). Structural basis for calmodulin as a dynamic calcium sensor. Structure.

[53] Shen M., Zhang N., Zheng S., Zhang W.-B., Zhang H.-M., Lu Z., Su Q.P., Sun Y., Ye K., Li X.-D. (2016). Calmodulin in complex with the first IQ motif of myosin-5a functions as an intact calcium sensor. Proc. Natl. Acad. Sci. USA.

[54] Montanarella A.F., Hundt N., Keim D., Venczel A., Zierhut F., Langnickel S., Graw A., Kröss M., Dietrich J., Saczko-Brack D. (2025). Cardiolipin membranes drive Myosin VI activation, oligomerization, and processive cargo transport. Proc. Natl. Acad. Sci. USA.

[55] Brito C., Sousa S. (2020). Non-muscle myosin 2A (NM2A): Structure, regulation and function. Cells.

[56] Kwon Y., Hofmann T., Montell C. (2007). Integration of phosphoinositide- and calmodulin-mediated regulation of TRPC6. Mol. Cell.

[57] Hokanson D.E., Laakso J.M., Lin T., Sept D., Ostap E.M. (2006). Myo1c binds phosphoinositides through a putative pleckstrin homology domain. Mol. Biol. Cell.

[58] Cao C., Zakharian E., Borbiro I., Rohacs T. (2013). Interplay between calmodulin and phosphatidylinositol 4,5-bisphosphate in Ca^2^+-induced inactivation of transient receptor potential vanilloid 6 channels. J. Biol. Chem..

[59] Mruk K., Farley B.M., Ritacco A.W., Kobertz W.R. (2014). Calmodulation meta-analysis: Predicting calmodulin binding via canonical motif clustering. J. Gen. Physiol..

[60] Agamasu C., Ghanam R.H., Xu F., Sun Y., Chen Y., Saad J.S. (2017). The interplay between calmodulin and membrane interactions with the pleckstrin homology domain of Akt. J. Biol. Chem..

[61] Andrews C., Xu Y., Kirberger M., Yang J.J. (2020). Structural aspects and prediction of calmodulin-binding proteins. Int. J. Mol. Sci..

[62] Oh B.-C. (2023). Phosphoinositides and intracellular calcium signaling: Novel insights into phosphoinositides and calcium coupling as negative regulators of cellular signaling. Exp. Mol. Med..

[63] O’Day D.H. (2026). The biological significance of calmodulin binding to lipids. Biology.

[64] Popov S.G., Krishna U., Falck J., Wilkie T.M. (2000). Ca2+/Calmodulin reverses phosphatidylinositol 3,4,5-trisphosphate-dependent inhibition of regulators of G protein-signaling GTPase-activating protein activity. J. Biol. Chem..

[65] Ishii M., Inanobe A., Kurachi Y. (2002). PIP _3_ inhibition of RGS protein and its reversal by Ca ^2+^/calmodulin mediate voltage-dependent control of the G protein cycle in a cardiac K^+^ channel. Proc. Natl. Acad. Sci. USA.

[66] Liu X., Wang Y., Weng Z., Xu Q., Zhou C., Tang J., Chen X.-Z. (2023). Inhibition of TRPP3 by calmodulin through Ca^2+^/calmodulin-dependent protein kinase II. Cell Insight.

[67] Liu X., Shu S., Billington N., Williamson C.D., Yu S., Brzeska H., Donaldson J.G., Sellers J.R., Korn E.D. (2016). Mammalian nonmuscle myosin II binds to anionic phospholipids with concomitant dissociation of the regulatory light chain. J. Biol. Chem..

[68] Park K.S., Kim S.H., Das A., Yang S.-N., Jung K.H., Kim M.K., Berggren P.-O., Lee Y., Chai J.C., Kim H.J. (2016). TLR3-/4-priming differentially promotes Ca^2+^ signaling and cytokine expression and Ca^2+^-dependently augments cytokine release in hMSCs. Sci. Rep..

[69] Salgado M., Sepúlveda-Arriagada V., Konar-Nié M., García-Robles M.A., Sáez J.C. (2025). Poly (I:C)-induced inflammation requires the activation of toll-like receptor 3/Ca^2+^/CaMKII/pannexin 1-dependent signaling. Theranostics.

[70] Lai Y.-C., Li C.-C., Sung T.-C., Chang C.-W., Lan Y.-J., Chiang Y.-W. (2019). The role of cardiolipin in promoting the membrane pore-forming activity of BAX oligomers. Biochim. Biophys. Acta (BBA)-Biomembr..

[71] Larrañaga-SanMiguel A., Bengoa-Vergniory N., Flores-Romero H. (2024). Crosstalk between mitochondria–ER contact sites and the apoptotic machinery as a novel health meter. Trends Cell Biol..

[72] Pays E. (2024). The Janus-faced functions of Apolipoproteins L in membrane dynamics. Cell. Mol. Life Sci..

[73] Balasubramanian K., Maeda A., Lee J.S., Mohammadyani D., Dar H.H., Jiang J.F., Croix C.M.S., Watkins S., Tyurin V.A., Tyurina Y.Y. (2015). Dichotomous roles for externalized cardiolipin in extracellular signaling: Promotion of phagocytosis and attenuation of innate immunity. Sci. Signal..

[74] Schulze H., Kolter T., Sandhoff K. (2009). Principles of lysosomal membrane degradation: Cellular topology and biochemistry of lysosomal lipid degradation. Biochim. Biophys. Acta (BBA)-Mol. Cell Res..

[75] Sorice M., Circella A., Cristea I.M., Garofalo T., Di Renzo L., Alessandri C., Valesini G., Degli Esposti M. (2004). Cardiolipin and its metabolites move from mitochondria to other cellular membranes during death receptor-mediated apoptosis. Cell Death Differ..

[76] Chai Z., Zhou Y., Hossain S., Huynh K., Hajimirzaei S., Zhang Y., Qi J., Zhang G., Li Z., Wei Y. (2025). Cardiolipin’s multifaceted role in immune response: A focus on interacting proteins. Front. Immunol..

[77] Frankenberg T., Kirschnek S., Häcker H., Häcker G. (2007). Phagocytosis-induced apoptosis of macrophages is linked to uptake, killing and degradation of bacteria. Eur. J. Immunol..

[78] Kågedal K., Johansson A.-C., Johansson U., Heimlich G., Roberg K., Wang N.S., Jürgensmeier J.M., Ollinger K. (2005). Lysosomal membrane permeabilization during apoptosis–involvement of Bax?. Int. J. Exp. Pathol..

[79] Bové J., Martínez-Vicente M., Dehay B., Perier C., Recasens A., Bombrun A., Antonsson B., Vila M. (2014). BAX channel activity mediates lysosomal disruption linked to Parkinson disease. Autophagy.

[80] Karch J., Schips T.G., Maliken B.D., Brody M.J., Sargent M.A., Kanisicak O., Molkentin J.D. (2017). Autophagic cell death is dependent on lysosomal membrane permeability through Bax and Bak. eLife.

[81] Dong H., Guo W., Zhou Z. (2023). BAX/MLKL signaling contributes to lipotoxicity-induced lysosomal membrane permeabilization in alcohol-associated liver disease. Autophagy.

[82] Jia J. (2026). Global cellular responses to lysosomal damage. Curr. Biol..

[83] Mao L., Li N., Guo Y., Xu X., Gao L., Xu Y., Zhou L., Liu W. (2013). AMPK phosphorylates GBF1 for mitotic Golgi disassembly. J. Cell Sci..

[84] Hansen M.D., Johnsen I.B., Stiberg K.A., Sherstova T., Wakita T., Richard G.M., Kandasamy R.K., Meurs E.F., Anthonsen M.W. (2017). Hepatitis C virus triggers Golgi fragmentation and autophagy through the immunity-related GTPase M. Proc. Natl. Acad. Sci. USA.

[85] Kim B.-H., Shenoy A.R., Kumar P., Bradfield C.J., MacMicking J.D. (2012). IFN-inducible GTPases in host cell defense. Cell Host Microbe.

[86] Haldar A.K., Saka H.A., Piro A.S., Dunn J.D., Henry S.C., Taylor G.A., Frickel E.M., Valdivia R.H., Coers J. (2013). IRG and GBP host resistance factors target aberrant, “non-self” vacuoles characterized by the missing of “self” IRGM proteins. PLoS Pathog..

[87] Fisch D., Pfleiderer M.M., Anastasakou E., Mackie G.M., Wendt F., Liu X., Clough B., Lara-Reyna S., Encheva V., Snijders A.P. (2023). PIM1 controls GBP1 activity to limit self-damage and to guard against pathogen infection. Science.

[88] Daussy C.F., Monard S.C., Guy C., Muñoz-González S., Chazal M., Anthonsen M.W., Jouvenet N., Henry T., Dreux M., Meurs E.F. (2021). The inflammasome components NLRP3 and ASC act in concert with IRGM to rearrange the Golgi apparatus during hepatitis C virus infection. J. Virol..

[89] Liu M., Duan Y., Dong J., Zhang K., Jin X., Gao M., Jia H., Chen J., Liu M., Wei M. (2024). Early signs of neurodegenerative diseases: Possible mechanisms and targets for Golgi stress. Biomed. Pharmacother..

[90] Lara-Reyna S., Clough B., Channell W.M., McCarthy C., Barlow J., Stanulović V.S., Hoogenkamp M., Roberts J., Marzullo B., Shenoy A.R. (2026). Golgi-localised Guanylate-binding protein 5 enhances glycolysis in macrophages. bioRxiv.

[91] Iwaniec M.O., Bott C.J., Casanova J.E. (2025). Loss of ARF5 impairs recovery after lysosomal damage. Front. Mol. Biosci..

[92] Mărunţelu I., Constantinescu A.-E., Covache-Busuioc R.-A., Constantinescu I. (2024). The Golgi apparatus: A key player in innate immunity. Int. J. Mol. Sci..

[93] Jena K.K., Mehto S., Nath P., Chauhan N.R., Sahu R., Dhar K., Das S.K., Kolapalli S.P., Murmu K.C., Jain A. (2020). Autoimmunity gene IRGM suppresses cGAS-STING and RIG-I-MAVS signaling to control interferon response. EMBO Rep..

[94] Yan X., Li J., Wang Y., Luo J., Yuan W., Xu X., Sheng Z., Aini M., Liu Y., Liu L. (2026). Activation of the cGAS–STING pathway mediated by IRGM1 contributes to the development of restrictive cardiomyopathy induced by cTnIR193H mutation. Genes Dis..

[95] De Tito S., Almacellas E., Yu D.D., Millard E., Zhang W., de Heus C., Queval C., Hervás J.H., Pellegrino E., Panagi I. (2025). ATG9A and ARFIP2 cooperate to control PI4P levels for lysosomal repair. Dev. Cell.

[96] Tang H., Wang Y., Wang S., Wu M., Lin S., Chen G. (2010). Atg1-mediated myosin II activation regulates autophagosome formation during starvation-induced autophagy. EMBO J..

[97] Kruppa A.J., Kendrick-Jones J., Buss F. (2016). Myosins, Actin and Autophagy. Traffic.

[98] Wang G., Wang T., Hu Y., Wang J., Wang Y., Zhang Y., Li F., Liu W., Sun Y., Yu B. (2020). NMMHC IIA triggers neuronal autophagic cell death by promoting F-actin-dependent ATG9A trafficking in cerebral ischemia/reperfusion. Cell Death Dis..

[99] Korobova F., Gauvin T.J., Higgs H.N. (2014). A role for myosin II in mammalian mitochondrial fission. Curr. Biol..

[100] Bond L.M., Brandstaetter H., Sellers J.R., Kendrick-Jones J., Buss F. (2011). Myosin motor proteins are involved in the final stages of the secretory pathways. Biochem. Soc. Trans..

[101] da Silva A.C.R., Kendrick-Jones J., Reinach F.C. (1995). Determinants of ion specificity on EF-hands sites: Conversion of the Ca^2+^/Mg^2+^ site of smooth muscle myosin regulatory light chain into a Ca^2+^-specific site. J. Biol. Chem..

[102] Rezaee F., Meednu N., Emo J.A., Saatian B., Chapman T.J., Naydenov N.G., De Benedetto A., Beck L.A., Ivanov A.I., Georas S.N. (2011). Polyinosinic:polycytidylic acid induces protein kinase D–dependent disassembly of apical junctions and barrier dysfunction in airway epithelial cells. J. Allergy Clin. Immunol..

[103] Hardigan T., Spitler K., Matsumoto T., Carrillo-Sepulveda M.A. (2015). Activation of Toll-like receptor 3 increases mouse aortic vascular smooth muscle cell contractility through ERK1/2 pathway. Pflüg. Arch.-Eur. J. Physiol..

[104] Pei Wong D.C., Xia Z., Shao N., Yeo J.Y., Yow I., Thivakar T., Salazar A.M., Liou Y.C., Low B.C., Ding J.L. (2023). T Natural Killer cell contractility and cytotoxicity is driven by TLR3 agonist-mediated TAZ cytoplasmic sequestration. bioRxiv.

[105] Greenberg M.J., Mealy T.R., Jones M., Szczesna-Cordary D., Moore J.R. (2010). The direct molecular effects of fatigue and myosin regulatory light chain phosphorylation on the actomyosin contractile apparatus. Am. J. Physiol. Integr. Comp. Physiol..

[106] Forster F., Paster W., Supper V., Schatzlmaier P., Sunzenauer S., Ostler N., Saliba A., Eckerstorfer P., Britzen-Laurent N., Schütz G. (2014). Guanylate binding protein 1–mediated interaction of T cell antigen receptor signaling with the cytoskeleton. J. Immunol..

[107] Ostler N., Britzen-Laurent N., Liebl A., Naschberger E., Lochnit G., Ostler M., Forster F., Kunzelmann P., Ince S., Supper V. (2014). Gamma interferon-induced guanylate binding protein 1 is a novel actin cytoskeleton remodeling factor. Mol. Cell. Biol..

[108] Bennison D.J., Chaudhary I., Chaudhuri D., Wong J.C.N., Punwatkar A., Biswas P., Stephenson M., Zhong Q., Kallemeijn W.W., Guenot M. (2026). GBP1 recruitment to actin-rich pedestals of extracellular Gram-negative bacteria promotes pyroptosis. EMBO J..

[109] Colonne P.M., Winchell C.G., Voth D.E. (2016). Hijacking host cell highways: Manipulation of the host actin cytoskeleton by obligate intracellular bacterial pathogens. Front. Cell. Infect. Microbiol..

[110] Kutsch M., González-Prieto C., Lesser C.F., Coers J. (2021). The GBP1 microcapsule interferes with IcsA-dependent septin cage assembly around *Shigella flexneri*. Pathog. Dis..

[111] Kellermeier J.A., Heaslip A.T. (2024). Myosin F controls actin organization and dynamics in *Toxoplasma gondii*. Mol. Biol. Cell.

[112] Blake T.C.A., Haase S., Baum J. (2020). Actomyosin forces and the energetics of red blood cell invasion by the malaria parasite Plasmodium falciparum. PLoS Pathog..

[113] Lim Y., Cho Y.-B., Seo Y.-J. (2024). Emerging roles of cytoskeletal transport and scaffold systems in human viral propagation. Anim. Cells Syst..

[114] Winter S.V., Niedelman W., Jensen K.D., Rosowski E.E., Julien L., Spooner E., Caradonna K., Burleigh B.A., Saeij J.P.J., Ploegh H.L. (2011). Determinants of GBP recruitment to toxoplasma gondii vacuoles and the parasitic factors that control it. PLoS ONE.

[115] Schulz O., Hanč P., Böttcher J.P., Hoogeboom R., Diebold S.S., Tolar P., e Sousa C.R. (2018). Myosin II synergizes with F-actin to Promote DNGR-1-dependent cross-presentation of dead cell-associated antigens. Cell Rep..

[116] Pizzuto M., Monteleone M., Burgener S.S., Began J., Kurera M., Chia J.R., Frampton E., Crawford J., Oliveira M., Kenney K.M. (2025). Cardiolipin inhibits the non-canonical inflammasome by preventing LPS binding to caspase-4/11. EMBO J..

[117] Prakash B., Renault L., Praefcke G.J., Herrmann C., Wittinghofer A. (2000). Triphosphate structure of guanylate-binding protein 1 and implications for nucleotide binding and GTPase mechanism. EMBO J..

[118] Curnow P., Hardy B.J., Dufour V., Arthur C.J., Stenner R., Hodgson L.R., Verkade P., Williams C., Shoemark D.K., Sessions R.B. (2020). Small-residue packing motifs modulate the structure and function of a minimal de novo membrane protein. Sci. Rep..

[119] Fontaine F., Lecordier L., Vanwalleghem G., Uzureau P., Van Reet N., Fontaine M., Tebabi P., Vanhollebeke B., Büscher P., Pérez-Morga D. (2017). APOLs with low pH dependence can kill all African trypanosomes. Nat. Microbiol..

[120] Matheoud D., Moradin N., Bellemare-Pelletier A., Shio M.T., Hong W.J., Olivier M., Gagnon É., Desjardins M., Descoteaux A. (2013). Leishmania evades host immunity by inhibiting antigen cross-presentation through direct cleavage of the SNARE VAMP8. Cell Host Microbe.

[121] Dingjan I., Paardekooper L.M., Verboogen D.R., von Mollard G.F., ter Beest M., Bogaart G.v.D. (2017). VAMP8-mediated NOX2 recruitment to endosomes is necessary for antigen release. Eur. J. Cell Biol..

[122] Dingjan I., Verboogen D.R., Paardekooper L.M., Revelo N.H., Sittig S.P., Visser L.J., von Mollard G.F., Henriet S.S., Figdor C.G., ter Beest M. (2016). Lipid peroxidation causes endosomal antigen release for cross-presentation. Sci. Rep..

[123] Qu Y., Sun Y., Yang Z., Ding C. (2022). Calcium ions signaling: Targets for attack and utilization by viruses. Front. Microbiol..

[124] Ñeco P., Fernández-Peruchena C., Navas S., Gutiérrez L.M., de Toledo G.Á., Alés E. (2008). Myosin II contributes to fusion pore expansion during exocytosis. J. Biol. Chem..

[125] Bhat P., Thorn P. (2009). Myosin 2 maintains an open exocytic fusion pore in secretory epithelial cells. Mol. Biol. Cell.

[126] Aoki R., Kitaguchi T., Oya M., Yanagihara Y., Sato M., Miyawaki A., Tsuboi T. (2010). Duration of fusion pore opening and the amount of hormone released are regulated by myosin II during kiss-and-run exocytosis. Biochem. J..

[127] Pays E. (2025). Apolipoprotein L2’s Role in Liver Fibrosis. Gastro Hep Adv..

[128] Chowdhury H.H., Zorec R. (2020). Exocytotic fusion pore under stress. Cell Stress.

[129] McArthur K., Whitehead L.W., Heddleston J.M., Li L., Padman B.S., Oorschot V., Geoghegan N.D., Chappaz S., Davidson S., Chin H.S. (2018). BAK/BAX macropores facilitate mitochondrial herniation and mtDNA efflux during apoptosis. Science.

[130] Zhao L., Liu P., Mao M., Zhang S., Bigenwald C., Dutertre C.-A., Lehmann C.H., Pan H., Paulhan N., Amon L. (2023). BCL2 Inhibition reveals a dendritic cell–specific immune checkpoint that controls tumor immunosurveillance. Cancer Discov..

[131] Vanhollebeke B., Pays E. (2006). The function of apolipoproteins L. Cell. Mol. Life Sci..

[132] Suzuki M., Youle R.J., Tjandra N. (2000). Structure of Bax: Coregulation of dimer formation and intracellular localization. Cell.

[133] Bleicken S., Jeschke G., Stegmueller C., Salvador-Gallego R., García-Sáez A.J., Bordignon E. (2014). Structural model of active Bax at the membrane. Mol. Cell.

[134] Zhang Z., Zhou L., Liao C., Lv F., Qi F., Du L., Pogmore J., Del Rosaio J., Andrews D.W., OuYang B. (2025). A high-resolution dimeric structure reveals a critical role of α-helix 9 in apoptotic Bax pore assembly in the mitochondrial membrane. bioRxiv.

[135] Mueller B.K., Subramaniam S., Senes A. (2014). A frequent, GxxxG-mediated, transmembrane association motif is optimized for the formation of interhelical Cα–H hydrogen bonds. Proc. Natl. Acad. Sci. USA.

[136] Decock M., Stanga S., Octave J.-N., Dewachter I., Smith S.O., Constantinescu S.N., Kienlen-Campard P. (2016). Glycines from the APP GXXXG/GXXXA transmembrane motifs promote formation of pathogenic Aβ oligomers in cells. Front. Aging Neurosci..

[137] González-Hernández M., Hoffmann M., Brinkmann C., Nehls J., Winkler M., Schindler M., Pöhlmann S. (2018). A GXXXA motif in the transmembrane domain of the Ebola virus glycoprotein is required for tetherin antagonism. J. Virol..

[138] Wu H., Cao R., Wen M., Xue H., OuYang B. (2022). Structural characterization of a dimerization interface in the CD28 transmembrane domain. Structure.

[139] Li E., Wimley W.C., Hristova K. (2012). Transmembrane helix dimerization: Beyond the search for sequence motifs. Biochim. Biophys. Acta (BBA)-Biomembr..

[140] Teese M.G., Langosch D. (2015). Role of GxxxG motifs in transmembrane domain interactions. Biochemistry.

[141] Khouili S.C., Priego E., Heras-Murillo I., Dunphy G., Mastrangelo A., Martínez-Cano S., Nuñez V., Rodrigo-Tapias M., Belinchón-García A., Garaude J. (2026). Mitochondrial complex I activity promotes antigen cross-presentation in dendritic cells. Sci. Immunol..

[142] Zha Z.-K., Deng C.-J., Shen L.-J., Liu L.-Z., Huang Y.-X., Li Y.-H., Lv L., Zhang K., Chen L.-S., Chen F.-E. (2026). Perforin-2 enhances antigen-specific CTL immune response by promoting cross presentation. Cell Death Dis..

